# The hierarchical organization of natural protein interaction networks confers self-organization properties on pseudocells

**DOI:** 10.1186/1752-0509-9-S3-S3

**Published:** 2015-06-01

**Authors:** Eugenia Galeota, Caius Gravila, Filippo Castiglione, Massimo Bernaschi, Gianni Cesareni

**Affiliations:** 1 Department of Biology, University of Rome 'Tor Vergata', Rome, Italy; 2 Istituto per le Applicazioni del Calcolo "M. Picone", CNR, Via dei Taurini 19, Rome, Italy; 3 Research Institute IRCSS "Fondazione Santa Lucia", Rome, Italy

## Abstract

**Background:**

Cell organization is governed and maintained via specific interactions among its constituent macromolecules. Comparison of the experimentally determined protein interaction networks in different model organisms has revealed little conservation of the specific edges linking ortholog proteins. Nevertheless, some topological characteristics of the graphs representing the networks - namely non-random degree distribution and high clustering coefficient - are shared by networks of distantly related organisms. Here we investigate the role of the topological features of the protein interaction network in promoting cell organization.

**Methods:**

We have used a stochastic model, dubbed ProtNet representing a computer stylized cell to answer questions about the dynamic consequences of the topological properties of the static graphs representing protein interaction networks.

**Results:**

By using a novel metrics of cell organization, we show that natural networks, differently from random networks, can promote cell self-organization. Furthermore the ensemble of protein complexes that forms in pseudocells, which self-organize according to the interaction rules of natural networks, are more robust to perturbations.

**Conclusions:**

The analysis of the dynamic properties of networks with a variety of topological characteristics lead us to conclude that self organization is a consequence of the high clustering coefficient, whereas the scale free degree distribution has little influence on this property.

## Background

Genes encode the amino acid sequence of their protein products that in turn assemble into organized cellular structures to perform specific functions that support cell physiology and organism development. In recent years the explosion of genome sequence data has enabled the investigation genome and proteome evolution and the formulation of hypotheses on the mechanisms creating diversity and fixation of the characteristics that we observe in extant organisms [1]. The paradigm used in comparative genomics and proteomics is that sequence and structure conservation is a sign of selective pressure and can be used to identify "functional" elements [2-4].

On the other hand gene products do not act in isolation but form a complex web whose specific links are important to determine phenotypes. Thus, at a different level, conservation of the specific physical and functional links among gene products might reveal the processes that have shaped "interactomes", the intricate web determining the formation of the functional complexes that we observe in the cell. Although the available experimental information about interactomes is not as complete and as accurate as the one on genomes, the recent completion of large protein interaction studies has offered an unprecedented amount of information about the protein web shaping cell organization [5-7]. We are now in the position to ask questions about conservation of protein interaction networks. At present, comparative interactome analysis has revealed a limited conservation when specific edges, representing physical interactions and connecting orthologous proteins are compared in interactomes of different model organisms [8]. On the other hand the topological properties of the graphs representing interactomes are similar in distantly related species and markedly different from typical random graphs. As a matter of fact, all experimental protein interaction networks display features, such as high clustering coefficient and scale-free degree distribution that are not found in random networks [9].

The question arises as to whether these topological properties are found in interactomes because they are the outcome of common evolutionary processes or rather they have been imposed on random or quasi-random networks by natural selection because they confer some selective advantage. As an example, the power-law degree distribution can be explained by a model based on growth and preferential-attachment, whereby new proteins preferentially link to highly connected proteins [10]. A second type of network models, known as hierarchical [11], can overcome some limits of "growth and preferential attachment" scheme and yield interactomes that are both scale free and characterized by a high clustering coefficient.

Alternatively these two topological characteristics may not be the result of any specific growth mechanism but rather they may have been selected in evolution because they confer to the cellular system some functional advantage. While some specific links among proteins may be conserved because they are part of functional modules, such as for instance signaling pathways, conservation of global graph properties are more likely to reflect some general properties of the cell.

We tested the hypothesis that some features of cell organization may be explained by molding of the cell interactome so that, under selective pressure, it acquires specific topological characteristics that are reflected in advantageous functional patterns in the cell.

To this purpose we use a cellular automaton model of a cell [12] and, starting from a random spatial distribution of proteins, we let the automaton evolve under interactions rules that are either determined experimentally, thereby mimicking the interactions that occur in the cell milieu, or selected at random. We next define a metric of cell self organization and we show that cellular automata evolving under experimentally-determined interaction-constraints reach a higher level of organization than those ruled by random interactions. Furthermore natural networks are more "robust", since they yield pseudocells, whose organization is not affected by small stochastic perturbations of the initial conditions of the system.

## Results and discussion

### Model from data

Graph representations of protein interaction networks are static and as such they are inadequate to model the highly dynamic protein interaction network inside the cell. To fill this methodological gap we have designed and implemented ProtNet a computer model that captures the discrete and stochastic nature of protein interactions [12]. ProtNet represents an in silico cell as a three-dimensional lattice in which molecular entities (proteins or protein complexes) can shift, rotate and form new complexes with their neighbors, or dissociate, depending on a set of interaction rules. Each lattice point of the automaton corresponds to a volume with linear size (~5 nm) comparable to the diameter of an average globular protein. The cell is filled with proteins with an occupancy (20%) compatible with the estimated crowding of proteins in the cell cytoplasm [13]. The whole procedure can be seen as a sort of "discrete molecular dynamics" applied to interacting proteins in a cell. We have used the ProtNet model to monitor the dynamic consequences of the global and local properties of protein interaction networks.

The biological protein interaction networks that provide the rules for the evolution of the cellular automaton are "yeast_net" and "human_net", representing the interactions in the yeast and human cells. Both networks have been obtained from the resource *mentha *[14]. This resource integrates all the published information curated by the IMEx [15] databases MINT [16], Intact [17], DIP [18], BIOGRID [19] and MatrixDB [20] and uses a ranking procedure, similar to the one implemented in WI-PHI [21], to offer human and yeast interactomes where each interaction is assigned a weight according to experimental support (See Additional file 1 and Additional file 2 for the list of Yeast and Human interactions). yeast_net includes the 4714 interactions with the highest weight. The complete list of experimental human interactions has been filtered by setting a weight threshold in order to have a number of high confidence interactions comparable with that of yeast_net. The two resulting networks both include 1890 protein species (nodes) and 4714 interactions (edges). To each natural network we associate a random network assembled according to Erdös-Rényi (ER) model [22]. In Table 1 we have reported the main topological characteristics of the networks that we have used in the simulations presented here.

**Table 1:** Topological metrics of the networks used for the simulations in ProtNet SSI (Self-Similarity Index), SFFI (Scale Free Fitting Index), APL (Average Path Length), ACC (Average Clustering Coefficient).

Network	MAX SSI	SFFI	APL	DIAMETER	TRANSITIVITY	ACC
Yeast	6.431	0.885	5.773	17	0.313	0.327
Human	2.051	0.927	4.511	13	0.070	0.090
Erdös-Rényi	1.342	0.325	4.863	10	0.002	0.002

**Figure 1:**
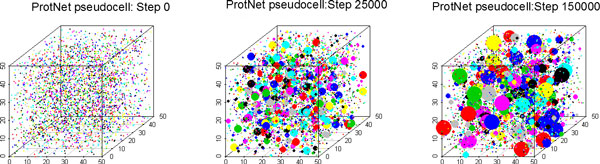
**ProtNet pseudocell dynamic organization**. The three boxes represent snapshots of the same pseudocell frozen at different steps during the simulation. In the first box protein monomers of different protein species are represented with different colors. After 5000 steps many small complexes are formed as a result of the diffusion and interaction dynamics. The size of the circles reflects the number of protein species in the complexes. Smaller spheres correspond to monomeric proteins. Complexes with the same number of protein species but different composition have different random colors. The third snapshot represents a psuedocell at equilibrium.

Irrespective of the interactions rules, starting from a random distribution of proteins in the space, the ProtNet cell evolves in time till it reaches a dynamic equilibrium state where the number of complexes of a given degree remains approximately constant (Figure 1). We call the cubic lattice whose organization evolves under the rules set by a protein interaction network a "pseudocell".

We have used such a model to answer the following questions about pseudocell organization.

a) Is the pseudocell obtained by using as input an experimental network different (i.e. more organized) than one originating from a network with an equivalent number of nodes connected at random?

b) If we perform independent simulations with the same input graph, how similar are the structures of the resulting pseudocells? Is the structure of pseudocells governed by experimental interaction rules more robust to perturbations introduced in the initial system settings? Does it make any difference if the input graph is a random or a natural network?

c) What are the network topological properties responsible for self-organization?

### Self-Similarity Index: a measure of cell organization

**Figure 2:**
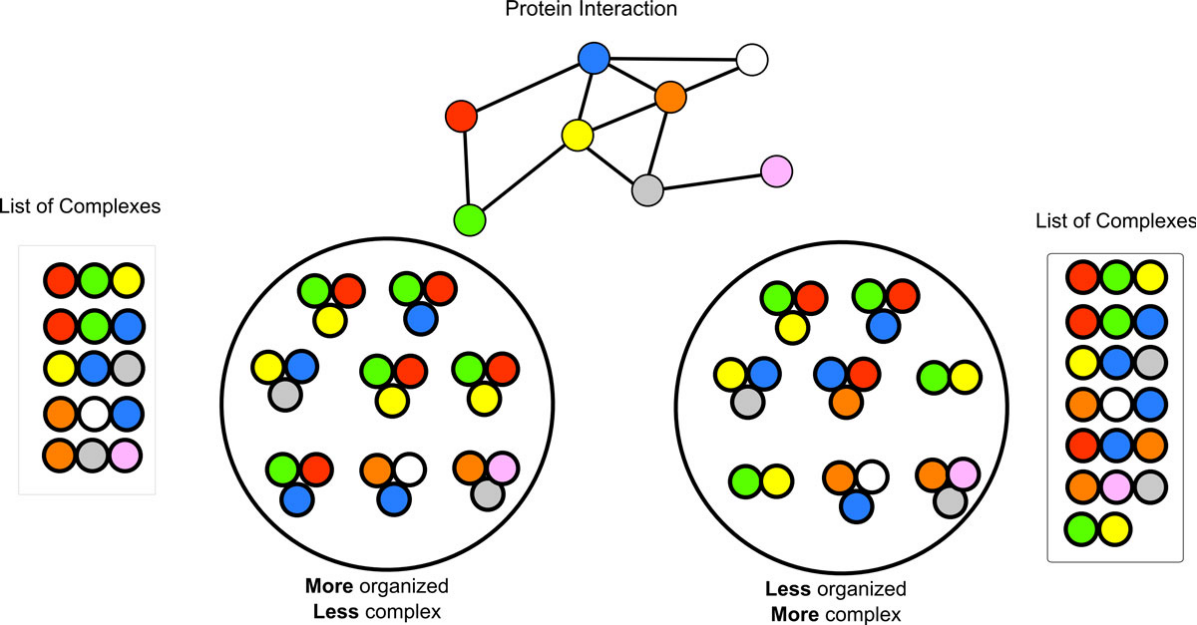
**Definition of coherence**. The cartoon schematically depicts our definition of coherence. The complexes that are formed in either pseudocell in the lower part of the figure are compatible with the interaction network at the top. However, the two pseudocells are fundamentally different because, while in the pseudocell on the left much fewer complexes are formed (see the list of complex species on the left side of the first pseudocell), in the one on the right each protein takes part in a larger variety of complexes as depicted in the list of complexes on the right side of the second pseudocell. Thus we conclude that the pseudocell on the left is simpler and therefore more organized than the one on the right. The similarity index between two complexes is obtained from the following formula $S.I.\left(C_{1},C_{2}\right)=\frac{{\left(\left|C_{1}\cap C_{2}\right|\right)}^{2}}{|\Delta \left(C_{1},C_{2}\right)|+1}$. The Self-Similarity Index of a pseudo-cell then can be computed as $SSI\left(C\right)=\frac{\sum_{i=1}^{n-1}max_{j=i+1}^{n}S.I\left(C_{i},C_{j}\right)}{n}$.

Interactomes obtained by integrating protein interaction data compiled from a variety of experimental techniques appear as intricate webs where each protein can deal with a large number of partners. This somewhat contrasts with the observation of organized leaving cells where any given protein tends to form one or a few specific protein complexes. Ribosomal proteins mostly assemble into ribosomes whereas proteasomal proteins are found preferentially in proteasomes. Even a simple interactome, such as the one in (Figure 2) can in principle support the formation of many complex types that are compatible with the rules established in the interaction graph. Nevertheless complexes formed in natural cells take a precise structure and dynamic organization emerging from both local and global properties of the interactome topology. In order to investigate if our model can simulate these self-organization properties we observed the evolution of the organization of our cellular automaton under the constraints of the interaction rules encoded in the interactome. We define a pseudocell as more organized if contains complexes that are more "coherent", that is if they are characterized by the presence of a cohesive subset of interacting proteins that, like a repeating pattern, can be found in many complexes. The rationale behind the idea of coherence and organization is more clearly explained in Figure 2. The two outlined pseudocells are both compatible with the interaction rules represented in the simple graph at the top. However, the complexes of different composition that are formed in the pseudocell at the left are fewer than the ones found at the right. Thus we define the first pseudocell as more organized. We introduce here a new metrics, the Self-Similarity Index (SSI), to quantify self-organization in pseudocells and compare pseudocells originated from different networks. The SSI is formally defined in the methods section. In a few words it defines how complex a pseudocell is. A pseudocell formed by identical large complexes has maximum SSI whereas a pseudocell containing a large variety of complexes of different composition has a small SSI.

**Figure 3:**
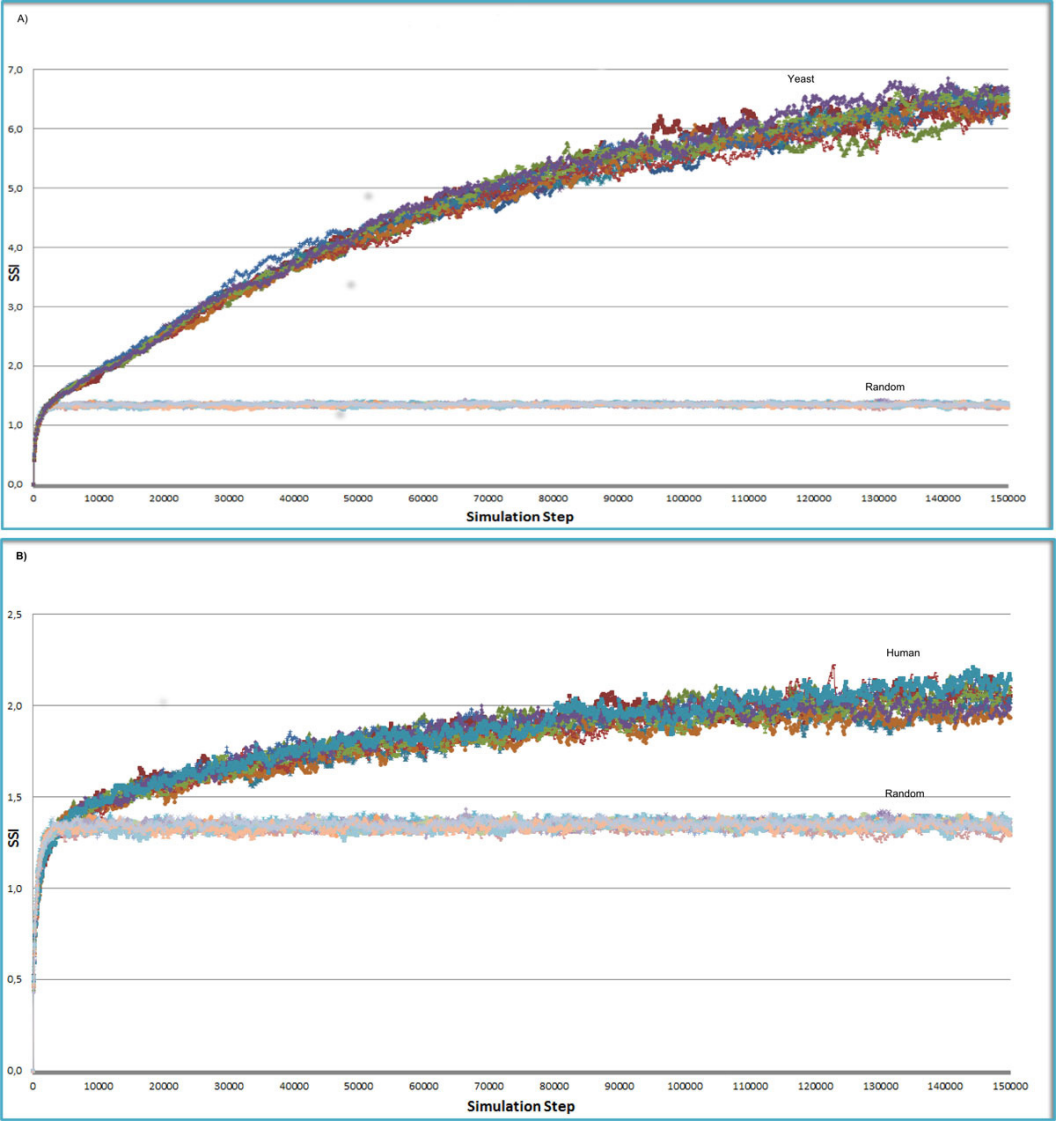
**Self-Similarity Index evolution**. We have measured the SSI values at different times during the evolution of a pseudocell. The first chart A) shows the evolution in time of the SSI for ten pseudocells that evolved following the interaction rules of the yeast protein interaction network and ten whose organization was evolved under the rules established by the random network. The second chart B) shows the results of a similar experiment where we used the human interactome.

Starting from proteins randomly distributed in the cell grid, Figure 3 shows the evolution in time of the SSI in pseudocells whose interactions are governed by the experimentally determined networks of *S. cerevisiae *(Figure 3 upper chart) and *Homo sapiens *(Figure 3 lower chart) or by random networks, with approximately the same number of nodes and edges. During the interaction and diffusion phases, small complexes start to aggregate till the pseudocell reaches an equilibrium state in which the emerged complex structure remains stable. The SSI rises comparably in both types of pseudocells, but after a few thousand simulation steps, when large complexes begin to form, the SSI of pseudocells governed by random networks reaches a plateau, while that of pseudocells governed by natural network interaction rules continues to grow. Notably the self organization properties of natural networks are observed in simulations of pseudocells covering a wide range of protein concentrations (Additional File 3). Consistently with our definition of SSI these results indicate that pseudocells governed by natural networks reach a higher level of organization, irrespective of protein concentration.

### Pseudocells are robust to perturbations

**Figure 4:**
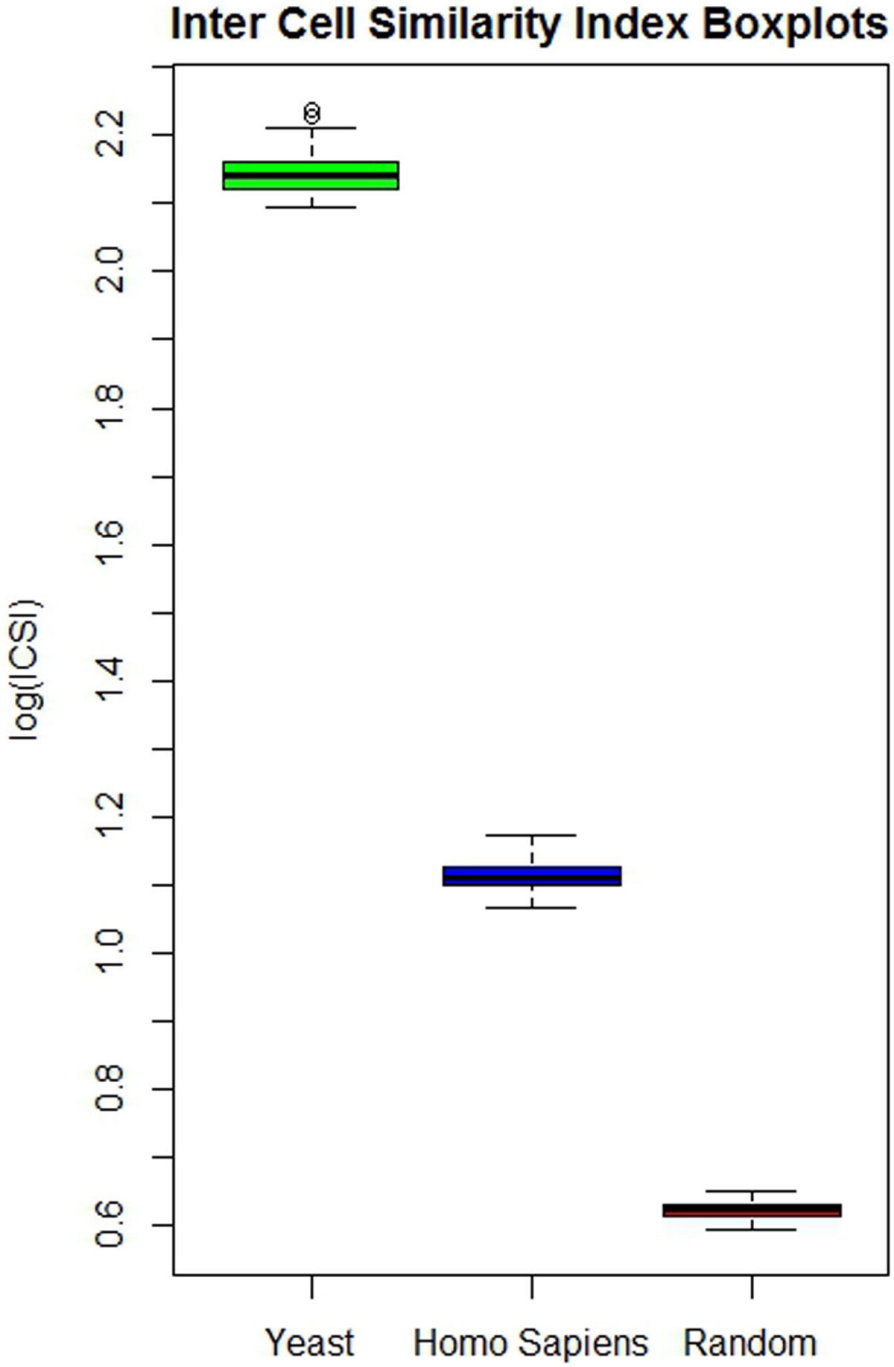
**Intercellular Similarity Index (ICSI) between pseudocells evolved under different starting conditions and different network rules**. For each of the three networks (yeast, human and random) we carried out 10 different simulations starting from different random distributions of the proteins in the pseudocell grid. We next calculated the ICSI between all the possible pairs of the 10 resulting pseudocells. The results are presented as boxplots.

An additional property of living cells is that of being robust to perturbations. In other words we expect a cell model to be able to recover organization, once it is perturbed, and to reach again a structure that is similar to that it had before perturbation. We modeled this property by measuring the similarity/diversity of cell organization when equilibrium is reached in the cell automaton, starting from different initial "cell configurations" (distribution of proteins inside the lattice). To compare different pseudocells we defined a second metric of cell organization that we call the Inter-Cells Similarity Index (ICSI). This metric (see methods) is similar to the SSI that we have defined to monitor cell organization. The difference being that ICSI measures the similarity of the ensemble of complexes that are formed in two different cells and not the diversity of the complexes in a cell. Figure 4 shows the pairwise ICSI values of ten different pseudocells for each of the three types of input networks (Yeast, Human and Random). Similarly to what observed in the case of a self organization of single cells, pseudocells that organize under the rules of the natural interactomes reach an organization that is similar in all the cells, irrespective of the initial distribution of the protein monomers.

### Self-Similarity Index at equilibrium depends on the topology of the underlying interactomes

Next we asked whether any topological property of the protein interaction network was responsible for the observed variation in the kinetic of pseudocell organization. To this end we generated families of networks with different, tuned, topological properties. Different algorithms have been proposed to generate networks with tunable parameters [23-27]. Most of them are based on the configuration model proposed in [24]. Here, in order to generate networks with tunable degree distribution and transitivity we used the algorithm developed by Volz [28]. We designed the network topology in three different settings:

• Designed networks whose degree distribution is taken from the yeast interactome but having transitivity coefficient in the interval 0[1] with steps of 0.1.

• Designed networks whose degree distribution is that of an Erdös-Rényi random network with transitivity coefficient in the interval 0[1] with steps of 0.1.

• Designed networks whose degree distribution is that of the human natural interactome with transitivity coefficient in the interval 0[1] with steps of 0.1.

**Figure 5:**
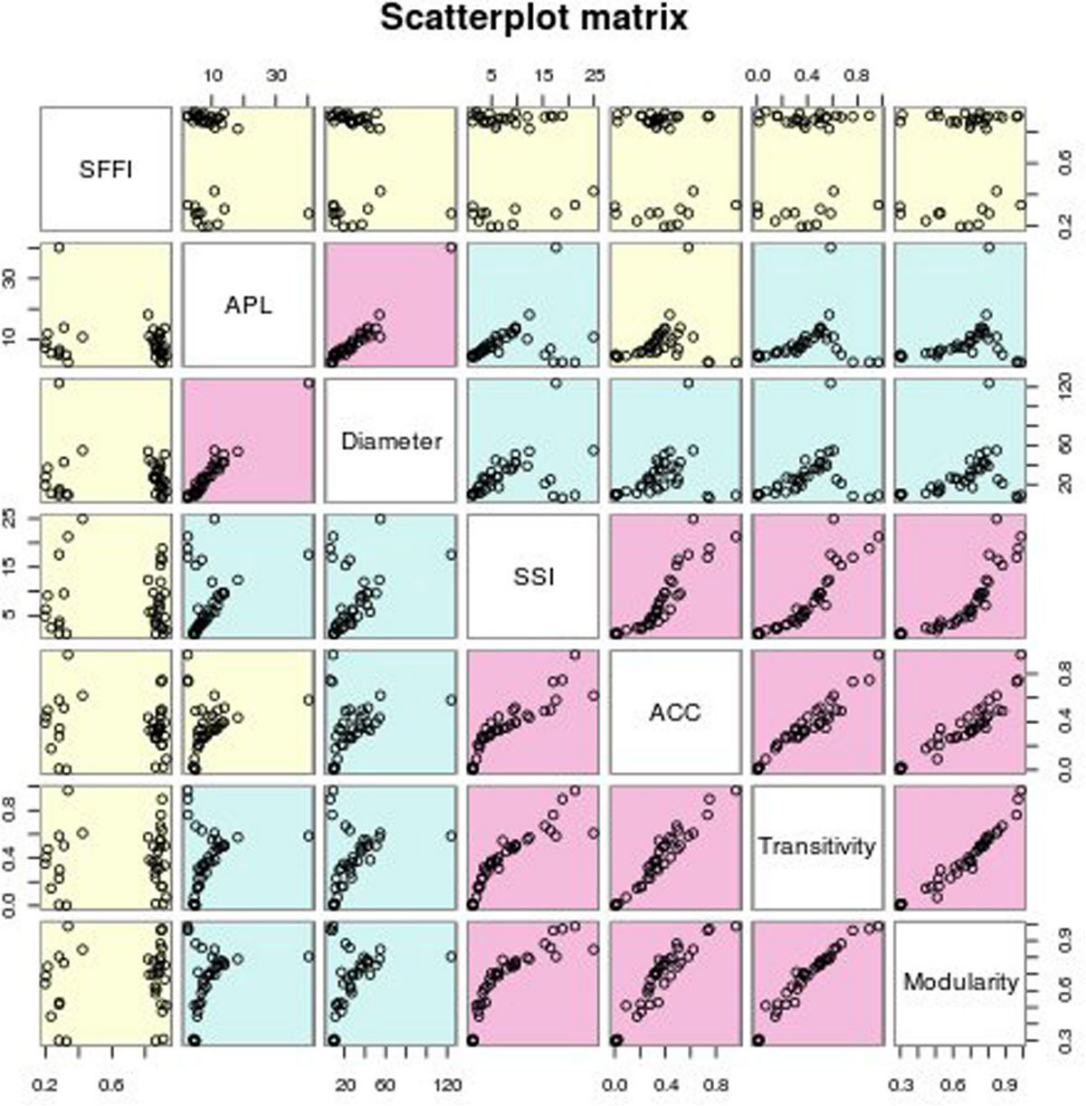
**Scatterplot Matrix of the linear dependencies between topological metrics**. The scatter-plot matrix shows all pair-wise correlations between the topological metrics measured for our networks: scale-free fitting index (SFFI), average path length (APL), Diameter, Self-Similarity Index (SSI), Average Clustering Coefficient (ACC), Transitivity and modularity. Each small chart represents the correlation between the metrics described in the corresponding diagonal elements. A linear relationship between all pair-wise combinations of SSI, ACC, Transitivity and Modularity is clearly evident. Also APL and Diameter are linearly correlated.

Table in Additional file 4 reports the topological characteristics of the described networks (respectively VolzYeast, VolzRandom and VolzHuman) together with the values of the Yeast and Human natural interactomes and an Erdös-Rényi network. These networks with designed topological properties, namely with different degree distributions and transitivity coefficients, were used as input for ProtNet. After 100000 steps the equilibrium SSI was measured. Notably we observed that increasing values of the transitivity coefficient corresponded to increased SSI values. To describe such relationship, we modeled a multiple linear regression equation where the SSI was associated to the different topological metrics of the initial networks. Our model is defined by the following regression equation:

$$SSI_{i}=\beta_{0}+\beta_{1}SFFI_{i}+\beta_{2}API_{i}+\beta_{3}DIAMETER_{i}+\beta_{4}ACC_{i}+\beta_{5}TRANSITIVITY_{i}+\beta_{6}MODULARITY_{i}+\varepsilon_{i}$$

where the dependent variables are the scale free fitting index [29] (SFFI), average path length (APL), diameter, average clustering coefficient [30] (ACC) (collective dynamics of small world networks), transitivity [31,32]. The dispersion matrix in Figure 5 shows all pair-wise combinations of the variables. A linear relationship between two or more independent variables indicates redundancy in explaining the dependent variable. The matrix describes linear relationships between transitivity, clustering coefficient and modularity and between average path length and diameter. F-test following regression allowed us to determine the redundant variables and remove them from the model. Regression results show a linear relationship between the transitivity coefficient (associated to the hierarchical modularity) and the SSI, while other network metrics such as the degree distribution, the average path length or the modularity were either redundant or non-significant. The adjusted R-squared value, used to measure the goodness of the fit, indicates that about the 80% of the SSI is explained by the transitivity coefficient. Further details about multiple linear regression is available in Additional file 2.

## Conclusions

The available experimental information on protein interaction networks reveals a poor conservation of the specific interactions between ortholog proteins in different model organisms. This is not surprising and could be explained by a high functional redundancy of some of the interactions occurring in a cell. In other words, aside from those few interactions that are necessary to form very specific complexes, many of the remaining interactions revealed by the experimental methods that are currently used to draw the interactome could be inessential but contribute in a redundant way to cell organization. For instance a protein that needs to perform its function near the cytoskeleton may find different ways to achieve its spatial localization by interacting with different partners linked to the cytoskeleton. On the other hand all interactomes described so far are characterized by graph topological properties that are conserved in distantly related species.

Here we have asked whether any of the topological features of protein interaction networks could be responsible for the ability of cells to reach a robust organization. To this end we have used a cell automaton model that simulates a cell space where interactions between proteins can occur. Although in principle the model can represent cells where proteins are present at different concentrations and interact with different affinities, here we focus on the topological properties of the network and we use a simple model where proteins are all present at the same concentration and interact with the same "strength".

By defining a new metrics of cell organization the Self-Similarity Index, we have shown that starting form a random distribution of protein monomers in a cell lattice, at each time step, protein complexes begins to form and the pseudocell starts organizing its structure. Interestingly the pseudocells that evolve under the rules of a natural protein interaction network reach a higher level of organization when compared to cells evolved under the interaction rules imposed by a random network.

Since the interactomes differ from random network in their topological properties we have asked which topological property is responsible for the ability of natural cells to reach a robust organization. By assembling modified networks with tailored degree distribution and clustering coefficients we have been able to show that the clustering coefficient explains more than 80% of the dependence of the Self-Similarity Index on topological properties. Thus, we propose that the clustering coefficient of the protein interaction network of natural cells has been fixed by natural selection to confer self organizing properties on the cell interactome.

## Methods

### ProtNet

ProtNet [12] is a cell automaton that permits the simulation and analysis the dynamic interactions occurring in a cell lattice under a set of interaction rules The simulator iterates for a number of time steps following the interaction rules contained in an input graph in which the edges link interacting proteins. A probability of forming and breaking a bond at each time step is associated to each pair of proteins. The algorithm runs the following steps:

• An empty lattice containing a given number of cubic sites is created. The linear dimension of the lattice is given as input to ProtNet.

• A single randomly oriented monomeric protein can occupy a lattice site. The number of proteins for each molecular species is fixed at the beginning of the simulation. Protein monomers are distributed randomly in the lattice cells.

Each simulation step is composed of two phases leading to a change in the cell configuration:

Interaction phase: The entire grid is visited site-by-site. If the site is occupied, the protein in it can make a connection to proteins occupying neighboring sites, thus forming a new complex. Alternatively an existing bond can be broken thus disrupting an existing complex. Binding is stochastic and the probability of forming a complex (p_on_) is related to the association rate constant (k_on_) of the interacting protein pair. After association a complex moves as a single entity. Similarly a binding between two proteins can break down with a dissociation probability (p_off_) related to their dissociation rates. Since kinetic constants describing the association and dissociation of all pairs of proteins are not currently available such probability has been set to an arbitrary value that is the same for all the proteins as suggested by [12].

Diffusion phase: A second site by site visit is carried out. If the site is occupied, the protein and the complex it belongs to are first rotated and then translated. Complexes or proteins can rotate by 90 degrees around their center of mass in a randomly chosen direction. Rotations are rigid, that is, in multi protein structures, the whole complex undergoes a rotation and there is no torsion. For multi protein complexes the probability of rotation is inversely proportional to their diameter. During the rotation and translation proteins occupying target sites can be recursively moved with a probability that is a function of the ratio between the masses of the hitting and hit protein/complex.

As the diffusion coefficient of a molecule in a dilute solution increases linearly with the inverse of the radius, monomers have the highest probability of moving to one of the neighbouring sites at each time step. As a consequence complexes diffuse more slowly. In addition we assumed that all proteins have identical diffusion coefficients.

Proteins in ProtNet have six binding sites, thus a single protein can bind to, at most, six other partners. To prevent the formation of very large complexes, each protein species can participate in a given complex only once.

For each simulation step ProtNet produces a list of the complexes in the reference pseudo-cell, a dynamic and stochastic representation of the information contained in static protein interaction graphs.

### Experimental settings

For our experiments the three-dimensional pseudocell linear dimension has been fixed to 50. Thus the whole lattice contains 125000 sites. The lattice is filled with proteins such that the occupancy is 20%. The interaction rules for natural networks are obtained from the human and yeast interactomes. The probability of creating a bond is set to 0.7 for all the protein pairs, while the probability of breaking a bond is set to 0.002. We have carried out simulations for 150000 steps on natural and random pseudocells. A simulation step, with a lattice containing 125000 sites at a protein concentration of 20%, takes 0.03 seconds.

To each natural network we associate a random network obtained by the Erdös-Rényi (ER) model [22] denoted as G(N, p). The method starts with N vertices and randomly links two nodes with probability p. In order to have the same number of edges of the natural networks we set $p=\frac{\frac{m}{N\left(N-1\right)}}{2}$ where *m *is the desired number of edges and *N *is the number of proteins in the network. We set (m = 4714 and N = 1890).

### Network construction

The input used in ProtNet is a list of binary interactions sorted by relevance score [14]. yeast_net is a network extracted from *mentha *[14]. The complete list of interactions was filtered in order to minimize false-positives and use only interactions described by more than one method. The resulting network is composed of 1890 protein species and 4714 interactions.

human_net, similarly to yeast_net is a network extracted from *mentha*, and containing a ranked list of human protein interactions that was limited to a size comparable to that of yeast_net.

We associate a node to each protein species in the list and draw an edge for each experimentally reported interaction. The interactomes used for our experiments can be formally represented as undirected unweighted graphs.

### Network analysis

To calculate and compare the topological properties of the different networks we used Cytoscape [33], a software platform for the visualization of molecular interaction networks extended with the NetworkAnalyzer plug-in [34]. From the plethora of available metrics we considered Clustering coefficient [30], Transitivity [31], Modularity [35], Connectivity and Degree distribution [36], Average path length, Diameter and Scale-free fitting index [29].

### Self-Similarity Index

In order to analyze the similarity between complexes we have introduced a measure called Self-Similarity Index. For our purpose we define a complex as a set of interacting proteins. Let $C_{1}$ and $C_{2}$ be the set of distinct protein species in two complexes. Their similarity index is defined as:

$$S.I.\left(C_{1},C_{2}\right)=\frac{{\left(\left|C_{1}\cap C_{2}\right|\right)}^{2}}{|\Delta \left(C_{1},C_{2}\right)|+1}$$

where Δ is the symmetric difference of the two complexes. The intersection between the two complexes is raised to the second power to give more importance to larger complexes.

Let $C=\left\{C_{1},C_{2},\ldots C_{n}\right\}$ be the list of complexes within a pseudocell, we define the Self-Similarity Index of the pseudocell as follows:

• We first generate a similarity matrix consisting of n rows and n columns as in Additional file 5.

• Each entry (i,j) of the similarity matrix contains the value of the Similarity Index (S.I.) between the complexes C_i _and C_j_.

• Then the Self-Similarity Index can be computed using the following formula:

$$SSI\left(C\right)=\frac{\sum_{i=1}^{n-1}max_{j=i+1}^{n}S.I\left(C_{i},C_{j}\right)}{n}$$

In other words the Self-Similarity Index of a pseudo-cell C is the mean value of the best similar pairwise similarities of the complexes in C.

### Inter-Cells Similarity Index (ICSI)

Given the definition of the similarity index between two complexes it is possible to compare the complex composition of different pseudo-cells and measure their similarity value.

Let $A=\left\{A_{1},A_{2},\cdots \;,A_{n}\right\}$ and $B=\left\{B_{1},B_{2},\cdots \;,B_{m}\right\}$ be the list of complexes of two pseudo-cells A and B. The Inter-cells Similarity Index can be computed as follows:

First generate a similarity matrix with n rows and m columns (Additional file 6). Then the algorithm proceeds as follows:

• Select the two complexes with the largest Similarity Index

• Store their Similarity Index into a List L

• Remove from the matrix the row and the column associated to those complexes

• Repeat the previous three steps until either columns or rows are finished

In other words at each step the algorithm finds the best matching complexes and computes their Similarity Indexes. The Inter-Cells Similarity Index (ICSI) is then computed as the mean value of the Similarity Indexes stored into the list.

### Generation of perturbed random networks

The comparison of different types of pseudo-cells to identify the topological metrics responsible for self-organization is based on the use of different initial networks, with tunable topological parameters as input for ProtNet. Different algorithms are available in the literature to generate random networks with tunable parameters [23-27]. Most of them are based on the configuration model proposed by Molloy and Reed which produces a random graph with a prescribed degree sequence. In the configuration model each node has an assigned potential number of edges called stubs. Edges are created randomly choosing two nodes with free stubs. An algorithm used to generate random networks with tunable degree distribution and transitivity is given in [28]. It is based on two mechanisms known as preferential attachment and dynamic growth. The input values are the number of nodes, a list of degrees to assign to nodes and the desired transitivity value. The algorithm works as follows:

• Start initializing all nodes with a degree drawn from the degree list.

• A starting node v_o _is randomly selected from the list of nodes

• Neighbors are matched in the following way:

• Form a list called PotentialTriads of all the nodes at distance 2 from the current node vi

• For each node in PotentialTriads form a connection with the current node with a probability depending on the desired Transitivity coefficient.

• If no neighbors were selected from PotentialTriads, randomly select a new node to add to the network.

The network grows until all nodes have neighbors.

## List of abbreviation used

SSI - Self-Similarity Index

ICSI - Inter-cells Similarity Index

ACC - Average clustering coefficient

APL - Average path length

SFFI - Scale free fitting index

## Competing interests

The authors declare that they have no competing interests.

## Authors' contributions

EG collected the data, carried out simulations, analyzed the results and drafted the manuscript.

CG contributed with statistical analysis

GC conceived the project supervised its development and wrote the manuscript

FC and MB provided computational expertise

## Supplementary Material

Additional file 1 **Yeast network**. The file contains the list of couples of interacting proteins filtered from the mentha "Yeast" interactome. Click here for file

Additional file 2 **Human network**. The file contains the list of pairs of interacting proteins filtered from the ***mentha ***"Homo Sapiens" interactome. Click here for file

Additional file 3 **SSI as a function of protein occupancy**. Simulations were carried out using either the yeast_net network or a random network at the indicated protein occupancies. The SSI was computed for 150000 simulation steps. Click here for file

Additional file 4 **Table of the networks with prescribed topological metrics**. The file contains the table with the topological metrics of the random interactomes obtained by perturbing transitivity coefficient and degree distribution. Click here for file

Additional file 5 **Similarity Matrix containing Similarity Indexes of the complexes of a pseudo-cell**. Click here for file

Additional file 6 **Similarity Matrix containing Similarity Indexes of the complexes of two different pseudocells A and B**. Click here for file

## Declarations

Publication of this article has been funded by ERC grant 322749-DEPTH.

This article has been published as part of *BMC Systems Biology *Volume 9 Supplement 3, 2015: Proceedings of the Italian Society of Bioinformatics (BITS): Annual Meeting 2014: Systems Biology. The full contents of the supplement are available online at http://www.biomedcentral.com/bmcsystbiol/supplements/9/S3.
