# Supplementary figures and images for: The hierarchical organization of natural protein interaction networks confers self-organization properties on pseudocells
Source: BMC Syst Biol. 2015 Jun 1;9(Suppl 3):S3. doi: 10.1186/1752-0509-9-S3-S3 (PMC4464023; doi:10.1186/1752-0509-9-S3-S3)

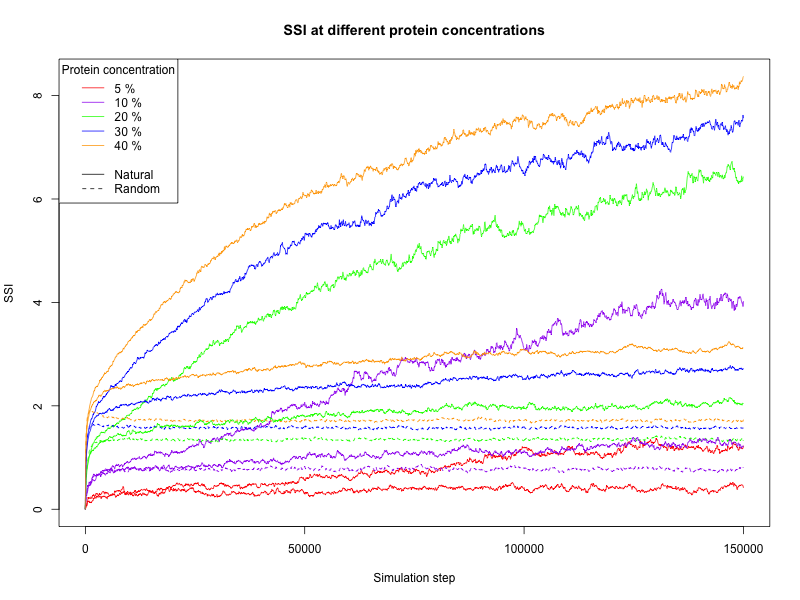

Supplement: Additional file 3 — SSI as a function of protein occupancy. Simulations were carried out using either the yeast_net network or a random network at the indicated protein occupancies. The SSI was computed for 150000 simulation steps. [file 1752-0509-9-S3-S3-S3.png]
