# Supplementary material for: The hierarchical organization of natural protein interaction networks confers self-organization properties on pseudocells
Source: BMC Syst Biol. 2015 Jun 1;9(Suppl 3):S3. doi: 10.1186/1752-0509-9-S3-S3 (PMC4464023; doi:10.1186/1752-0509-9-S3-S3)
Supplement: Additional file 4 — Table of the networks with prescribed topological metrics. The file contains the table with the topological metrics of the random interactomes obtained by perturbing transitivity coefficient and degree distribution. [file 1752-0509-9-S3-S3-S4.docx]

Topological metrics of natural interactomes and random perturbed networks

| **Network** | **Self-Similarity Index** | **Scale-free Fitting Index** | **Average Path Length** | **Diameter** | **Transitivity** | **Average Clustering Coefficient** | **Modularity** |
| --- | --- | --- | --- | --- | --- | --- | --- |
| VolzYeast1 | 1.304 | 0.864 | 4.297 | 10 | 0.016 | 0.024 | 0.300 |
| VolzYeast2 | 2.330 | 0.903 | 5.653 | 17 | 0.158 | 0.212 | 0.476 |
| VolzYeast3 | 3.276 | 0.861 | 6.792 | 27 | 0.234 | 0.263 | 0.586 |
| VolzYeast4 | 4.141 | 0.859 | 8.257 | 26 | 0.277 | 0.280 | 0.624 |
| VolzYeast5 | 5.968 | 0.848 | 9.732 | 28 | 0.366 | 0.337 | 0.701 |
| VolzYeast6 | 5.798 | 0.826 | 11.034 | 45 | 0.384 | 0.330 | 0.697 |
| VolzYeast7 | 7.875 | 0.877 | 10.560 | 36 | 0.454 | 0.351 | 0.732 |
| VolzYeast8 | 9.757 | 0.850 | 13.228 | 42 | 0.502 | 0.397 | 0.777 |
| VolzYeast9 | 12.384 | 0.818 | 18.123 | 54 | 0.574 | 0.436 | 0.790 |
| VolzYeast10 | 16.526 | 0.901 | 6.846 | 25 | 0.632 | 0.501 | 0.857 |
| VolzYeast11 | 18.832 | 0.902 | 2.503 | 6 | 0.895 | 0.749 | 0.971 |
| VolzRandom1 | 1.382 | 0.280 | 4.901 | 9 | 0.007 | 0.010 | 0.301 |
| VolzRandom2 | 2.554 | 0.233 | 5.400 | 11 | 0.146 | 0.177 | 0.446 |
| VolzRandom3 | 3.089 | 0.281 | 5.753 | 12 | 0.229 | 0.279 | 0.517 |
| VolzRandom4 | 3.873 | 0.284 | 6.570 | 15 | 0.301 | 0.344 | 0.529 |
| VolzRandom5 | 4.885 | 0.198 | 7.284 | 19 | 0.353 | 0.393 | 0.643 |
| VolzRandom6 | 6.357 | 0.201 | 8.888 | 28 | 0.405 | 0.441 | 0.689 |
| VolzRandom7 | 9.157 | 0.213 | 11.862 | 37 | 0.474 | 0.497 | 0.745 |
| VolzRandom8 | 9.583 | 0.310 | 13.919 | 43 | 0.505 | 0.518 | 0.766 |
| VolzRandom9 | 17.600 | 0.281 | 40.239 | 124 | 0.586 | 0.582 | 0.805 |
| VolzRandom10 | 24.910 | 0.423 | 10.802 | 55 | 0.609 | 0.620 | 0.848 |
| VolzRandom11 | 21.281 | 0.335 | 2.461 | 9 | 0.967 | 0.959 | 0.987 |
| VolzHuman1 | 1.383 | 0.909 | 4.282 | 10 | 0.016 | 0.024 | 0.310 |
| VolzHuman2 | 2.712 | 0.898 | 6.023 | 19 | 0.165 | 0.206 | 0.520 |
| VolzHuman3 | 3.551 | 0.868 | 7.361 | 26 | 0.320 | 0.265 | 0.610 |
| VolzHuman4 | 4.654 | 0.917 | 7.483 | 23 | 0.338 | 0.284 | 0.668 |
| VolzHuman5 | 4.948 | 0.870 | 9.062 | 33 | 0.374 | 0.312 | 0.702 |
| VolzHuman6 | 7.162 | 0.887 | 11.864 | 42 | 0.546 | 0.350 | 0.786 |
| VolzHuman7 | 8.678 | 0.888 | 12.257 | 35 | 0.493 | 0.377 | 0.755 |
| VolzHuman8 | 9.659 | 0.918 | 13.714 | 51 | 0.504 | 0.396 | 0.753 |
| VolzHuman9 | 11.963 | 0.894 | 10.096 | 39 | 0.554 | 0.456 | 0.801 |
| VolzHuman10 | 15.387 | 0.892 | 5.007 | 21 | 0.670 | 0.491 | 0.882 |
| VolzHuman11 | 17.044 | 0.895 | 2.555 | 8 | 0.763 | 0.734 | 0.962 |
| NaturalYeast | 6.431 | 0.885 | 5.7332 | 17 | 0.314 | 0.3276 | 0.7115 |
| NaturalHuman | 2.051 | 0.927 | 4.511 | 13 | 0.070 | 0.090 | 0.510 |
| Erdős–Rényi | 1.342 | 0.325 | 4.860 | 10 | 0.003 | 0.003 | 0.297 |
