# Supplementary material for: The hierarchical organization of natural protein interaction networks confers self-organization properties on pseudocells
Source: BMC Syst Biol. 2015 Jun 1;9(Suppl 3):S3. doi: 10.1186/1752-0509-9-S3-S3 (PMC4464023; doi:10.1186/1752-0509-9-S3-S3)
Supplement: Additional file 5 — Similarity Matrix containing Similarity Indexes of the complexes of a pseudo-cell. [file 1752-0509-9-S3-S3-S5.docx]

|  | **C_1_** | **C_2_** | **C_3_** | **…** | **C_n-1_** | **C_n_** |
| --- | --- | --- | --- | --- | --- | --- |
| **C_1_** |  | S.I.(C_1_,C_2_) | S.I.(C_1_,C_3_) | … | S.I.(C_1_,C_n-1_) | S.I.(C_1_,C_n_) |
| **C_2_** |  |  | S.I.(C_2_,C_3_) | … | S.I.(C_2_,C_n-1_) | S.I.(C_2_,C_n_) |
| **C_3_** |  |  |  | … | S.I.(C_3_,C_n-1_) | S.I.(C_3_,C_n_) |
| **…** |  |  |  | … | … | … |
| **C_n-1_** |  |  |  |  |  | S.I.(C_n-1_,C_n_) |
| **C_n_** |  |  |  |  |  |  |
