# Supplementary material for: The hierarchical organization of natural protein interaction networks confers self-organization properties on pseudocells
Source: BMC Syst Biol. 2015 Jun 1;9(Suppl 3):S3. doi: 10.1186/1752-0509-9-S3-S3 (PMC4464023; doi:10.1186/1752-0509-9-S3-S3)
Supplement: Additional file 6 — Similarity Matrix containing Similarity Indexes of the complexes of two different pseudocells A and B. [file 1752-0509-9-S3-S3-S6.docx]

|  | **B_1_** | **B_2_** | **…** | **B_m-1_** | **B_n_** |
| --- | --- | --- | --- | --- | --- |
| **A_1_** | S.I.(A_1_,B_1_) | S.I.(A_1_,B_2_) | … | S.I.(A_1_,B_m-1_) | S.I.(A_1_,B_m_) |
| **A_2_** | S.I.(A_2_,B_1_) | S.I.(A_2_,B_2_) | … | S.I.(A_2_,B_m-1_) | S.I.(A_2_,B_m_) |
| **…** | ... | ... | … | … | … |
| **A_n-1_** | S.I.(A_n-1_,B_1_) | S.I.(A_n-1_,B_2_) | ... | S.I.(A_n-1_,B_m-1_) | S.I.(A_n-1_,B_m_) |
| **A_n_** | S.I.(A_n_,B_1_) | S.I.(A_n_,B_2_) | ... | S.I.(A_n_,B_m-1_) | S.I.(A_n_,B_m_) |
